# Structural insights into the DNA-binding specificity of E2F family transcription factors

**DOI:** 10.1038/ncomms10050

**Published:** 2015-12-03

**Authors:** Ekaterina Morgunova, Yimeng Yin, Arttu Jolma, Kashyap Dave, Bernhard Schmierer, Alexander Popov, Nadejda Eremina, Lennart Nilsson, Jussi Taipale

**Affiliations:** 1 Department of Biosciences and Nutrition, Karolinska Institutet, SE 141 83 Stockholm, Sweden; 2 European Synchrotron Radiation Facility, Division of Experiments, 38 000 Grenoble, France; 3 Department of Biochemistry and Biophysics, Stockholm University, SE 106 91, Sweden; 4 Genome-Scale Biology Research Program, Faculty of Medicine, University of Helsinki, PO Box 63, FI-00014 Helsinki, Finland

## Abstract

The mammalian cell cycle is controlled by the E2F family of transcription factors. Typical E2Fs bind to DNA as heterodimers with the related dimerization partner (DP) proteins, whereas the atypical E2Fs, E2F7 and E2F8 contain two DNA-binding domains (DBDs) and act as repressors. To understand the mechanism of repression, we have resolved the structure of E2F8 in complex with DNA at atomic resolution. We find that the first and second DBDs of E2F8 resemble the DBDs of typical E2F and DP proteins, respectively. Using molecular dynamics simulations, biochemical affinity measurements and chromatin immunoprecipitation, we further show that both atypical and typical E2Fs bind to similar DNA sequences *in vitro* and *in vivo*. Our results represent the first crystal structure of an E2F protein with two DBDs, and reveal the mechanism by which atypical E2Fs can repress canonical E2F target genes and exert their negative influence on cell cycle progression.

## Abstract (web summary)

The E2F family of transcription factors controls many important cellular processes. Here, the authors determine the structure of an atypical E2F that contains two DNA binding domains, and propose a mechanism of action for these atypical E2Fs.

## Graphical abstract

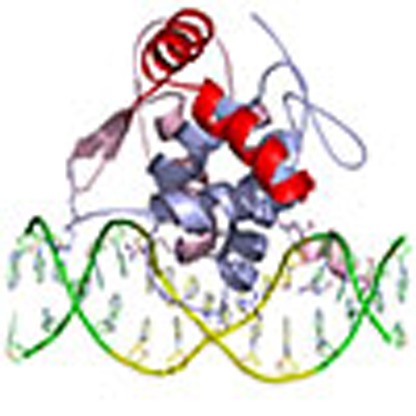

E2F transcription factor family proteins encompass a wide range of functions in cell cycle regulation, cell differentiation, DNA stress response and apoptosis^1^,^2^,^3^,^4^. The family is divided into two subfamilies: E2Fs 1–3 are activators of transcription, whereas E2Fs 4–8 act as repressors (Fig. 1a). Whereas the E2F proteins 1–6 bind to DNA preferentially as heterodimers with the related DP proteins DP1 and DP2, the two most recently discovered members of the E2F family, E2F7 and E2F8, are ‘atypical‘, because they contain two distinct DNA-binding subdomains. They also lack the pocket protein-binding domain found in all other E2Fs, and thus are not regulated by the canonical cyclin-dependent kinase/retinoblastoma protein pathway^5^.

Genetic evidence indicates that the atypical E2Fs regulate the same processes as the typical E2Fs. For example, the placental defect caused by loss of both E2F7 and E2F8 is rescued by the loss of the activator E2F3a^6^,^7^. However, initial analyses of the binding specificities of typical and atypical E2Fs has suggested that the proteins bind to different sites. The typical E2Fs in complex with DP proteins have been reported to bind to a canonical E2F site 5′-TTTC[CG]CGC-3′ (refs 8, 9, 10) and they have also proposed to differ in their binding specificity^11^,^12^,^13^,^14^,^15^. In contrast, more recent work by several investigators have suggested that the typical and atypical E2Fs can bind to the same core sequence 5′-GGCGGG-3′ (refs 16, 17, 18).

So far the only structural information of E2F-DNA complexes comes from the heterodimeric complex of E2F4 with the DP2 protein bound to the canonical E2F DNA sequence 5′-TTTCGCGCGGTTT-3′ (ref. 19; PDB entry 1CF7). The DNA-binding fragment of E2F8 is different from that of E2F4; it is comprised of two DBDs (DBD1 and DBD2) that show limited similarity to each other in amino-acid sequence (33.8% identity). The DBDs are connected by an 82-amino-acid linker. Because the similarity in amino-acid sequence between E2F4, DP2 and E2F8 is relatively low, the existing structure cannot be effectively used to model the differences in DNA binding between typical and atypical E2Fs.

To address this problem, we expressed an E2F8 protein fragment containing both predicted DBDs (residues Gln110–Ile350) in *Escherichia coli*, crystallized the purified protein and solved its structure in complex with its preferred DNA sequence 5′-TTTTTGGCGGGAAAA-3′ (ref. 17).

## Results

### Crystal structure of E2F8

**Figure 1:**
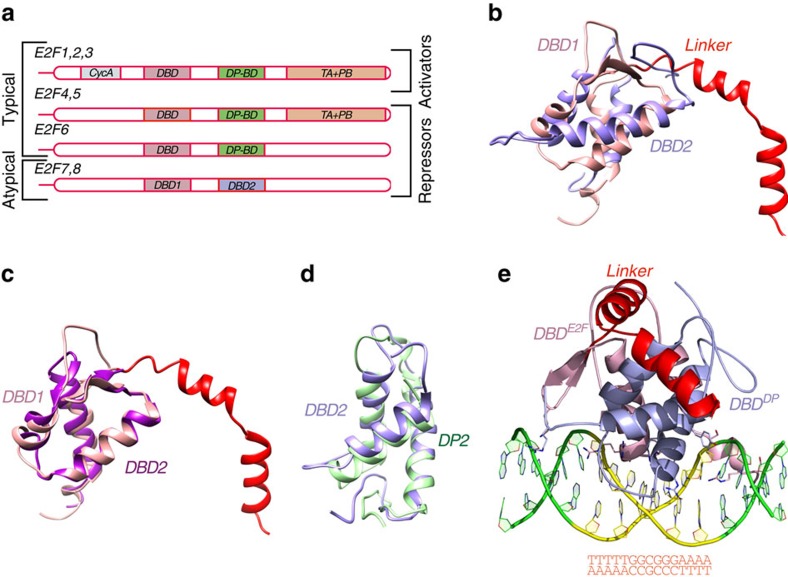
Structure of E2F8. (**a**) Schematic representation of the structural organization of E2F transcription factors. Key: CycA: cyclin A-binding domain; DBD: DNA-binding domain; DP-BD: DP-binding domain; TA+PB: transactivation and pocket protein-binding domains. Note that the typical E2Fs have DP-binding domains, which are replaced by a second DBD in the atypical E2Fs. (**b**) Superimposition of E2F8 DBD1 (pink) and DBD2 (blue) (r.m.s.d.=7.8 Å); the linker between the two DBDs is in red. (**c**,**d**) Superimpositions of E2F8 DBD1 (pink) to E2F4 (magenta) (r.m.s.d.=1.36 Å; PDB ID 1CF7) and E2F8 DBD2 (blue) to DP2 (green) (r.m.s.d.=1.9 Å; PDB ID 1CF7). The 23 amino acids of the linker close to DBD1 are folded into two α-helices, whereas the remaining 53 amino acids connected to DBD2 are disordered. Note the high similarity between the domains. (**e**) Structure of the E2F8 protein containing DBD1 (DBD^E2F^, pink) and DBD2 (DBD^DP^, blue) bound to a 15-base pair DNA fragment (green and yellow). Residues responsible for the motif recognition are presented as ball-and-stick models and coloured by atom (carbon: chain colour; nitrogen: blue; oxygen: red). The sequence of the DNA fragment is also shown.

Analysis of the structure derived from a cubic and hexagonal crystal forms revealed that both DBDs are composed of three α-helixes and a small antiparallel β-sheet (Fig. 1b). Structural alignment showed that in spite of the fact that both DBDs belong to the winged-helix family, their structural arrangement is rather different (root mean squared deviation (r.m.s.d.)=7.8 Å; Fig. 1b). However, the comparison of E2F8's first and second DBDs to E2F4 and DP2, respectively, revealed striking structural similarity between them (r.m.s.d.=2.7 and 1.9 Å, respectively), despite the sequence identity being only 39.7 and 27.5% (Fig. 1c,d; Supplementary Fig. 1). The E2F8 DBDs 1 and 2 are thus referred to as DBD^E2F^ and DBD^DP^ hereafter (Fig. 1e).

The largest difference between E2F8 and E2F4/DP2-DNA complexes was observed in the inter-domain interaction area. The interface area between the E2F8 subdomains is approximately two times the size of that between E2F4 and DP2 (2,606 versus 1,238 Å^2^). About 40% of the residues from both the DBD^E2F^ and DBD^DP^ are involved in the formation of the inter-domain contact, whereas only 19.5 and 28.4% of E2F4 and DP2 residues, respectively, participate in the corresponding interaction (Supplementary Fig. 1b; Supplementary Table 1). The difference in the interaction area is in large part due to the contribution of residues from the long linker between the E2F8 DBDs. Of the 82 residues that comprise the linker, 23 are resolved in the structure, forming two α-helixes that wrap around the surface of the DBD^E2F^ and DBD^DP^ on the opposite side of the bound DNA.

Investigation of shared crystal-packing interactions between the cubic and hexagonal crystals of E2F8 revealed a symmetric interaction surface between two E2F domains. The interface area of the contact was relatively large, 1,147 Å^2^ (Supplementary Fig. 2a), suggesting that the interaction is potentially biologically relevant. No corresponding interaction was identified in the E2F4/DP2 structure. The interface contacts are formed by symmetrically related helices α1 between conserved Ser_112_, Glu_115_, Ala_127_ and Arg_128_ residues from one E2F8 molecule and identical residues of the symmetry-related molecule. The interaction is additionally supported by hydrophobic interactions between Leu_121_, Cys_122_, His_123_ and Phe_125_. Sequence alignment of E2F8 with E2F7 (Supplementary Fig. 2b) showed that in spite of the high conservation of the residues maintaining helix α1, both Glu_115_ and His_123_ of E2F8 are replaced with Gln residues in E2F7, which makes putative heterodimer or a E2F7 homodimer less stable than a E2F8 homodimer. The crystal structure and additional ATR-Fourier transform infrared (FTIR) and circular dichroism (CD) experiments (Supplementary Fig. 2c,d) indicate that dimerization is compatible with DNA binding. The orientation of the dimer partners also indicates that DNA looping or bending is required for binding of a homodimeric form of E2F8 to DNA.

Analysis of the DNA shape in the E2F8 and E2F4/DP2 complexes, using the programme Curves+ (ref. 20), revealed broadly similar effects of the proteins on the DNA structure (Supplementary Fig. 3a,b). The total bend of E2F8-bound DNA is 1.4 Å larger (8.9 Å/7.5 Å), whereas the average opening (2.2 Å/3.5 Å) and the average twist (9.4°/35.8°) are smaller than those of E2F4/DP2-bound DNA.

### Protein/DNA interactions

**Figure 2:**
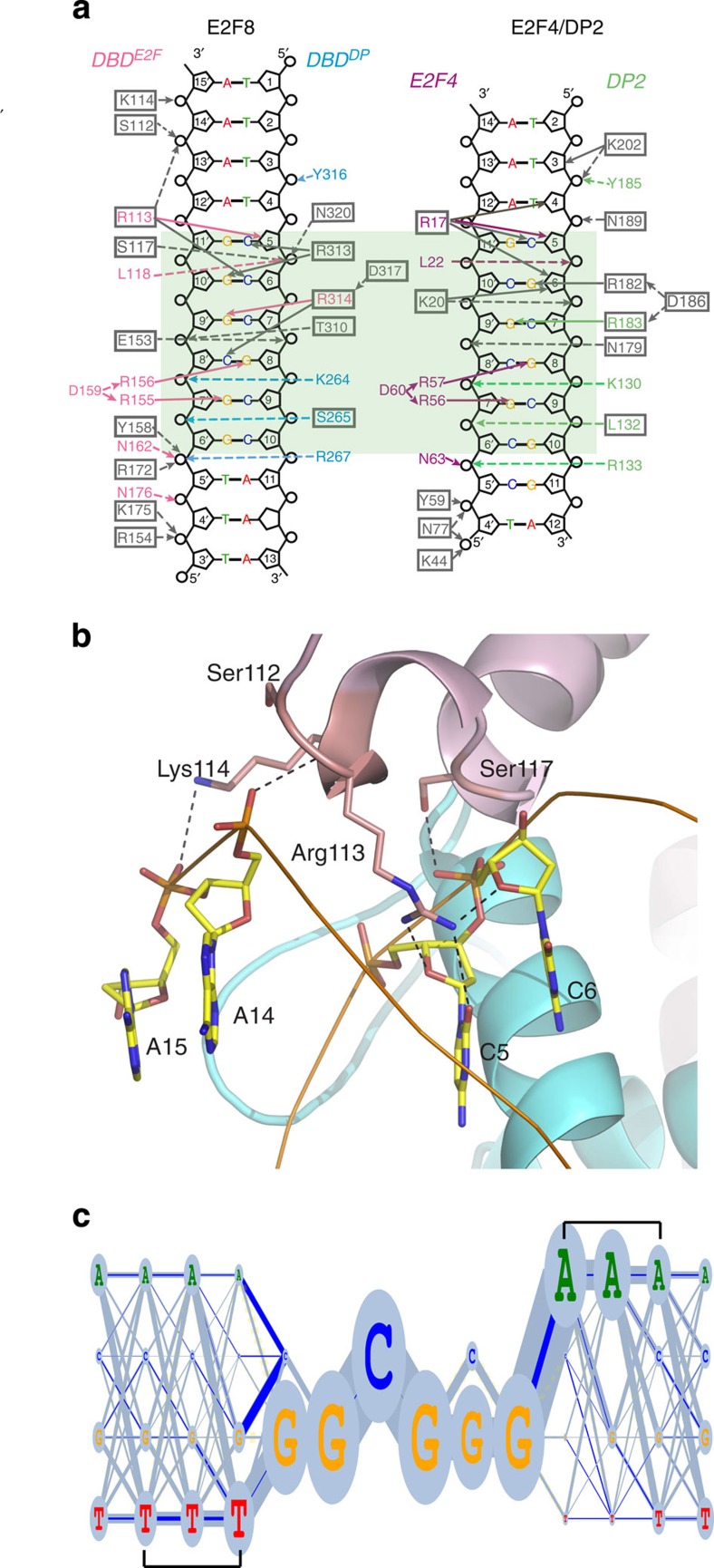
E2F8/DNA interactions. (**a**) Schematic representation of interactions formed between E2F8 and DNA (left panel) and E2F4/DP2-DNA (right panel). Equivalent contacts are highlighted by colouring, and amino acids that make different contacts are indicated by boxes. Residues belonging to the E2F and DP domains of E2F8 are in pink and blue, and residues of E2F4 and DP2 are magenta and green, respectively. Dashed lines represent interactions with phosphates in the DNA backbone and solid lines represent interactions to deoxyribose or to the bases. The light-green box indicates the core specificity region. (**b**) Contacts between E2F8 and DNA that are involved in recognition of a narrow minor groove. Residues and bases are presented as ball-and-stick models and coloured by atom (carbon: yellow; nitrogen: blue; oxygen: red). Hydrogen bond contacts are indicated by dashed lines, and their distance is indicated in italic. (**c**) DNA-binding preference of E2F8 identified using HT-SELEX. A first-order Markov (dinucleotide) model of the specificity of E2F8 is shown in a ‘riverlake' logo. Ovals indicate frequency of bases at each position, and width of the lines between them the frequency of the corresponding dinucleotide. Dark-blue lines indicate the extent to which a dinucleotide is more frequent than what is predicted from the mononucleotide frequencies. Note that AA and TT dinucleotides are preferred before the 5′-TGGCGGGA-3′ core sequence (brackets).

The contacts created with DNA by the E2F4/DP2 complex and the two DBDs of E2F8 are very similar (Fig. 2a). In both complexes, the DNA recognition helices of the E2F and DP domains are tightly packed into the same major groove. The E2F domains of E2F4 and E2F8 bind to DNA in an almost identical manner, forming contacts with C_5_ C_6_ G_8_ of one strand and G_7_′ of the complementary strand, and in addition, form several backbone contacts with both strands. Some differences in backbone contacts are observed; the E2F domain of E2F8 has two backbone contacts formed by Arg_154_ and Arg_172_ (Supplementary Fig. 4a,b), which are not present in E2F4, and it also lacks a contact analogous to that formed by Lys_44_ in E2F4 because this residue is replaced with Leu_143_ (Fig. 2a). In the DP2 and the E2F8 DP domains, a minor difference is observed in the geometry of the backbone contact formed by Tyr_316_. The orientation of the aromatic ring of Tyr_316_ in E2F8 is well supported by Ile_312_ and Phe_308_, which in DP2 are replaced by Arg and Gln, respectively (Supplementary Figs 1 and 4d).

The inclusion of 3–5 A/T base pairs on the flanks contributes strongly to the narrowing of the minor grooves in both structures^21^. The narrowing of the minor grooves on the DBD^E2F^ side of the DNA motif is recognized by an arginine in both the E2F4/DP2 and E2F8 structures. In the case of E2F8, Arg_113_ inserts into the minor groove, making contact to the oxygen of C_5_ and sugar of C_5_ and C_6_. The adjacent Lys_114_, in turn, contacts the backbone on the opposite side of the minor groove (Fig. 2b; Supplementary Fig. 4c). Furthermore, the positions of the phosphates of the DNA backbone on both sides of the minor groove are recognized by the main-chain oxygen of Ser_112_ and by the side chain of Ser_117_. Together, these contacts lock the Arg_113_ in place, leading to a preference of a narrow minor groove 5′ to the core 5′-TGGCGGGA-3′ motif. The resulting specificity towards three to four consecutive A or T bases is clearly visible in the E2F8 site obtained from SELEX experiments (Fig. 2c; see also ref. 17). The minor groove flanking the other side of the 5′-TGGCGGGA-3′ motif is also occupied by Lys_175_ from the E2F domain (Supplementary Fig. 4e). No similar contacts were observed in the E2F4/DP2 complex, despite the lysine being conserved.

**Figure 3:**
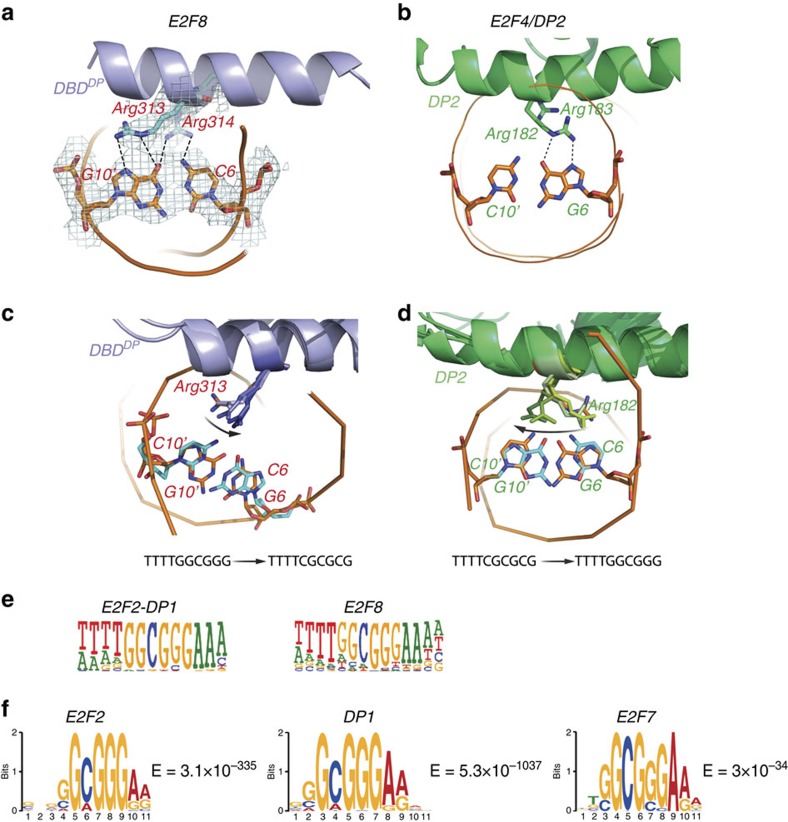
Atypical and typical E2Fs prefer similar motifs. (**a**) Close-up view of the contacts between the E2F8 DBD^DP^ arginines 313 and 314 and the DNA base pair G10′-C6 (C opposite to the capitalized G in tggcgGga). (**b**) The corresponding contact between DP2 and DNA in the E2F4/DP2-DNA complex. Note that the bound DNA sequence is different, and the arginine 182 of DP2 makes contact to a guanine on the opposite strand of DNA compared to that recognized by the corresponding Arg_313_ of E2F8. (**c**,**d**) Molecular dynamics simulations of the E2F8 DBD^DP^ bound to 5′-TTTTTCGCGCG-3′ (**c**) and the DP2 protein bound to 5′-TTTTGGCGGG-3′ (**d**). Five snapshots taken every 20 ns are shown. The original position of the Arg residue and the original base pair are coloured in orange. All following positions are coloured in progressively darkening color. The mutated base pairs are coloured in blue. Note that upon change of the underlying DNA sequence, the arginine moves (arrow) to the position observed in the other crystal, suggesting that atypical and typical E2Fs can recognize the same set of sequences. (**e**) HT-SELEX analyses for E2F2/DP1 complex and E2F8 performed in this study reveal that a typical E2F/DP and an atypical E2F prefer sequences that are very similar to each other (note that the obtained E2F8 motif is very similar to that reported in ref. 17). (**f**) Typical and atypical E2Fs prefer similar sequences *in vivo*. The most enriched motifs from genomic sequences bound by E2F2 and DP1 in ChIP-exo experiments performed in this work are virtually identical to a motif that is enriched by E2F7 in ChIP-seq (data from ref. 44). MEME *E*-values of the motifs are also shown.

The major difference between the structures is that in contrast to E2F4/DP2, E2F8 binding to DNA is not symmetrical, due to differences between DNA recognition by DP2 and the E2F8 DP domain. The contacts made by the recognition motif R_313_R_314_L_315_Y_316_D_317_ of the E2F8 DP domain are different from those formed by the corresponding motif of DP2. The first Arg residue 313 of this motif in E2F8 DBD^DP^ forms specific contacts with the oxygen atom of guanine G_10_′ (Fig. 3a; Supplementary Fig. 4f). However, the corresponding arginine (182) in DP2 is directed to G_6_ on the other strand of DNA and forms two hydrogen bonds with oxygen and nitrogen atoms of the base (Fig. 3b). As the G_10_′ is replaced by C_10_′ in the E2F4/DP2-DNA complex, the observed difference could either be caused by the different DNA sequences in the co-crystals or by different amino-acid sequences of the proteins.

### Molecular dynamics

To address whether the difference in DNA recognition by Arg_313_ is inherent in the protein sequences, we first performed molecular dynamics simulation experiments using E2F4/DP2-DNA and E2F8-DNA structures. Simulating E2F8 in complex with DNA where its co-crystallized DNA sequence 5′-GGCGGG-3′ was replaced by the 5′-G**C**GCG**C**C-3′ sequence of the E2F4/DP2 crystal revealed that Arg_313_ of E2F8 moved to a position similar to that observed in the E2F4/DP2 crystal. Conversely, simulating the E2F4/DP2 heterodimer with the E2F8-DNA sequence 5′-GGCGGG-3′ revealed that the corresponding Arg in E2F4 moved to form a contact that was similar to that observed in E2F8. These results suggest that E2F8 and E2F4/DP2 proteins can recognize the same DNA sequences (Fig. 3c,d). To confirm that E2F8 prefers the sequence we used for crystallization, we compared the affinities of E2F8 with the two different core sequences using isothermal titration calorimetry (ITC). These experiments revealed a kDa of ∼260 nM for E2F8 site containing the 5′-GGCGGG-3′ core sequence (Supplementary Fig. 5). Affinity for the 5′-GCGCGCC-3′ core was below the detection limit for this method.

### DNA-binding motifs *in vitro* and *in vivo*

To directly address the DNA-binding specificities of the E2F family members, we performed SELEX experiments with E2F2 in the absence or presence of the DP1 protein. These results revealed that in the presence of DP1 protein, the motif-containing sequence of (T)5′-TTGGCGGGAA-3′(A) was preferred over the 5′-GGCGCC-3′ site bound by E2F homodimers, or the canonical 5′-GCGCGC-3′ E2F/DP site reported previously (Fig. 3e).

Finally, to assess the specificity of E2F proteins *in vivo*, we performed chromatin immunoprecipitation (ChIP)-exo experiments for E2F2 and DP1. We then performed motif-mining of peaks from these experiments and from an E2F7 ChIP-seq experiment from ref. 44. This analysis revealed that all proteins preferred essentially identical sequences (Fig. 3f), corresponding to the highest-affinity core sequence identified by SELEX. These results, together with our structural and molecular dynamics results indicate that the atypical E2Fs are capable of binding to sites recognized by the typical E2F/DP heterodimers.

## Discussion

In this work, we have determined the three-dimensional structure of a non-canonical E2F, E2F8, bound to its preferred DNA sequence. We found that the two DBDs of E2F8, DBD^E2F^ and DBD^DP^, are structurally highly similar to E2F4 and DP2 DBDs, respectively. The protein–DNA contacts are very similar between the E2F8 DBDs and those of E2F4 and DP2. However, the protein–protein contacts are divergent. Whereas the interaction between typical E2Fs and DP proteins is mediated by separate interaction domains, in E2F8, the linker between the DBDs strongly contributes to the inter-DBD interaction.

We also observed an interaction between symmetry-related molecules that involved helix α1 that potentially facilitates the dimerization of two E2F8 proteins. Such homo- and heterodimer formation between the atypical E2Fs E2F7 and E2F8 has been reported earlier^5^,^18^,^22^, but additional specific studies are needed to confirm the importance of the observed contacts in formation of atypical E2F dimers.

The two DBDs of E2F8 recognize a 5′-GGCGGG-3′ core sequence by inserting their helices α3 into the same major groove of DNA. In addition, the minor grooves from both sides of the sequence are narrowed by the inclusion of four consecutive A/T base pairs. The preference to the 5′ and 3′ flanking sequences is due to contacts formed by Arg_113_, Lys_114_ and Ser_117_, and Lys_175_ in the minor grooves, respectively (see also refs 19, 21).

The comparison of contacts found in DBD^E2F^-DNA and E2F4-DNA showed that they are mostly similar, with a few differences due to the difference in the DNAs used for crystallization and the amino-acid sequences of the proteins. To analyse the differences, we performed molecular dynamic simulations that showed that difference in contacts of a key arginine residue is due to differences in the DNA sequences used for crystallization. This result indicate that E2F8 and E2F4/DP2 can recognize the same DNA sequence consisting of a 5′-GGCGGG-3′ core sequence, with the E2F and DP -like domains binding to the GGC and GGG half-sites, respectively. The similarity in binding specificity between typical E2F/DP complexes and atypical E2Fs was validated using both *in vitro* selection (SELEX) and motif mining from ChIP-exo-enriched peaks.

In summary, through extensive structural and functional analyses of the E2F proteins, we have defined the DNA-binding specificities of E2F and E2F/DP complexes. These analyses have revealed that the two domains of the atypical repressor E2Fs correspond to the E2F and DP domains, which bind to DNA sites that are indistinguishable from those bound by typical E2F/DP heterodimers. Our results define the binding specificity of the E2F family of transcription factors, and reveal the mechanism by which E2F8 is capable of regulating the cell cycle by directly repressing target genes activated by the typical E2F/DP heterodimers.

## Methods

### Protein purification, crystallization and data collection

The human E2F8 (residues Gln_110_–Ile_350_) polypeptide was purified by affinity chromatography and gel-filtration chromatography based on the principles described in ref. 23. A complementary DNA encoding E2F8 DBD, containing N-terminal thioredoxin and a 6 × His-tag, optimized for expression in *E. coli* was purchased from GenScript and subcloned into the pETG-20A vector. The construct was verified by sequencing and expressed in Rosetta(DE3)pLysS *E. coli* strain (Millipore). The expression of recombinant protein was induced by addition of isopropyl-β-d-thiogalactopyranoside to 0.5 mM final concentration. Culture was grown overnight at 17 °C, harvested and lysed using immobilized metal-ion-affinity chromatography lysis buffer (50 mM Tris-Cl, 300 mM NaCl, 10 mM imidazole, 10% glycerol, pH 7.5). The protein purification was conducted on an ÄKTA Xpress system with His-Trap HP column (GE Healthcare) and a HiLoad 16/600 Superdex 200 gel-filtration column (GE Healthcare). The His-Trap HP column was equilibrated in 100 mM HEPES, 500 mM NaCl, 10% glycerol, 10 mM imidazole, 0.5 mM Tris(2-carboxyethyl)phosphine hydrochloride (TCEP), pH 7.5, and the thioredoxin-fused-His-tagged protein was eluted with 20 mM HEPES buffer containing 500 mM NaCl, 500 mM imidazole, 10% glycerol and 0.5 mM TCEP. The N-terminal thioredoxin 6 × His-tag was removed by incubation of pooled protein fractions with TEV protease overnight. The resulted E2F8 DBD protein solution was concentrated and applied onto the gel filtration column equilibrated in 20 mM HEPES buffer, pH 7.5, containing 150 mM NaCl, 5% glycerol and 0.5 mM TCEP. The flow-through containing the cleaved E2F8 DBD was collected and concentrated up to ∼10 mg ml^−1^. Purity of the protein was examined on SDS–polyacrylamide electrophoresis gel stained with Coomassie brilliant blue. The correct mass of the protein preparations was confirmed using matrix-assisted laser desorption/ionization time-of-flight mass spectrometry analysis (Mass spectrometry, Core facilities, University of Oulu, Finland).

The DNA fragments used in crystallization were obtained from Integrated DNA Technologies (BVBA, Belgium) as single-strand oligos and annealed in 10 mM Tris (pH 7.5) containing 150 mM NaCl and 1 mM EDTA. The purified E2F8 was first mixed with solutions of the DNA duplex at a molar ratio of 1:1.2 and after 15–20 min on ice subjected to the crystallization trials. An in-house developed crystal screening kit of different polyethylene glycols (PEGs) in addition to JBScreen Nuc-Pro HTS from Jena Bioscience were applied to complexes with DNAs of different length. Only complexes with DNAa and DNAb (5′-TTTTGGCGGGAAAAA-3′ and 5′-ATTTTTGGCGGGTTTG-3′, respectively) showed micro-crystals under several conditions containing PEG (6000), KCl and MgCl_2_. Further optimization of conditions allowed to obtain two types of crystals of cubic and hexagonal shapes. Cubic crystals were grown in sitting drops by the vapour diffusion technique at room temperature from 100 mM Hepes (pH 7.09) solution containing 8% (w/v) PEG (6000), 150 mM KCl, 2 mM MgCl_2_ and 5% PEG (200). Crystals were grown to full size (0.25 mm) overnight. Hexagonal crystals were obtained from 100 mM Hepes (pH 7.09) containing 4.8% PEG (4000), 120 mM ammonium sulfate and 5% of PEG (400). Those crystals reached full size (0.3 mm) in 1–2 weeks. The data were collected from both types of crystals at European Synchrotron Radiation Facility (Grenoble, France) from a single crystal on beam-line ID23-1 at 100 K and wavelength 0.9763 Å using the reservoir solution as a cryoprotectant. The data collection strategy was optimized with the programme BEST^24^. Data were integrated with the programme XDS^25^ and scaled with XSCALE. The cubic crystals diffract to 3.9 Å resolution only. They belong to the I23 space group with cell dimensions *a*=172.8 Å containing one molecule of complex in an asymmetric unit with 78.8% of solvent. Crystals of hexagonal shape diffracted slightly better, up to 3.07 Å resolution, and belonged to the hexagonal space group P3_2_2_1_ with cell dimensions *a*=98.8 Å, *c*=121.7 Å, also containing one molecule of complex per asymmetric unit and 75% solvent. Crystals of both symmetries showed merohedral twining with relatively small twinning fractions of 0.032 and 0.122 for I23 and P3_2_2_1_ crystals, respectively. Statistics of data collection are presented in Table 1.

### Structure determination and refinement

The initial phases of the E2F8 E2F domain model in both crystal forms were determined by molecular replacement using the programme Phaser^26^ in Phenix^27^ with the structure of E2F4 from the E2F4/DP2 complex (PDB entry 1CF7 (ref. 19)) as a search model. The sequence alignment of each DBD of E2F8 with E2F4 and DP2 (Supplementary Fig. 1) showed 34 and 30% identity, respectively. Thus, the first search was performed for the DBD^E2F^, then, the first solution was fixed and the DBD^DP^ was found. The rigid body and jelly-body refinement with REFMAC^28^ dropped original *R*-factors from 55% (57%) to 33% (36%). At this point the manual rebuilding of the model was done using COOT^29^. The resulting models were used to search for the solutions in two other data sets. The multi-crystal averaging between all three data sets was applied to improve the quality of the electron density. The standard TLS refinement with Phenix.refine was combined with two more cycles of multi-crystal averaging and Phenix_den.refine. Due to low resolution, only one data set was used to complete the refinement and build the final model. In all, 86.81% and 11.54% of residues were found in favoured and allowed regions of Ramachandran map. The refinement statistics are presented in Table 1.

### Molecular dynamics

Molecular dynamics simulations were performed for the following protein–DNA complexes: the E2F8 DBD^DP^ complexed with 5′-TTTTTCGCGCGAAAA-3′ and DP2 complexed with 5′-AAAAGGCGGGAAAA-3′ (PDB entry 1CF7). Models of the ‘mutants' were built manually by switching the cytosine and guanine bases in a CG base pair, without changing the protein structure. The CHARMM 36 forcefield^30^,^31^,^32^,^33^,^34^ and CHARMM program^35^, with the CHARMM interface to OpenMM^36^ to allow the use of NVIDIA graphical processing units, were used for all simulations. The starting structure was placed in a cubic solvent box with 8-nm side length containing water^37^ and sodium ions to neutralize the system. After energy minimization to relax initial strain, the systems were heated from 100 to 300 K over 8 ps followed by 12 ps simulation at constant pressure (1 bar) and constant temperature (300 K), with soft harmonic positional restraints on the protein and DNA atoms. In the subsequent 200 ns production runs using the graphical processing unit, the pressure and temperature were also maintained at 1 bar and 300 K, respectively, and the positional restraints were removed. Particle mesh Ewald summation was used to treat the long-range electrostatic interactions, using a 5th-order B-spline interpolation for the charge distribution on the 0.1-nm-spaced grid points, kappa=0.34. The same 0.9-nm cutoff was used for both the direct space part of the PME and for the van der Waals interactions, which were switched to zero from 0.8 to 0.9 nm, and the non-bond list was generated with a 1.1-nm cutoff. SHAKE^38^ was used to keep the lengths of all covalent X-H bonds fixed, allowing a time step of 2 fs. The structural analysis used the last 100 ns of the trajectories.

### Isothermal titration calorimetry

To determine affinities of the DNA motifs described above, ITC experiments were carried out using an ITC200 microcalorimeter (MicroCal Inc., Northampton, Massachusetts, USA) in PSF (Protein Science Facility at Karolinska Institute, Sweden), and GE Healthcare (Sweden). Binding isotherms of DNAs were measured by direct titration of protein to the cell containing DNA. The measurements were taken at 20 °C. Both protein and DNA were prepared in a buffer containing 20 mM HEPES pH 7.5, 150 mM NaCl, 5% glycerol and 0.5 M TCEP. To measure binding affinity, a solution of 0.1 mM protein was titrated to 0.012–0.016 mM solution of DNA. A total of 20 injections were made with 240 s between injections. All data were evaluated using the OriginPro 7.0 software package (Microcal) supplied with the calorimeter. The apparent dissociation constant *K*_d_, binding enthalpy ΔH and stoichiometry *n*, together with their corresponding s.d., were determined by a nonlinear least-squares fit of the data to standard equations for the binding using a model for one set of independent and identical binding sites as implemented in the package. The entropy and free energy of binding were obtained from the relation *ΔG=−*RT*lnK*_d_*=ΔH−TΔS*.

### HT-SELEX

The DBD sequences of E2F2, E2F8 and DP1 were cloned into N-terminal thioredoxin 6 × His bacterial expression vector (pETG-20A; Vincentelli *et al*., 2011) with either no additional affinity tag (DP1), a C-terminal streptavidin-binding peptide (E2F8), or 3 × FLAG (E2F2) tag by Gateway LR reaction (Invitrogen). The recombinant proteins were expressed in Rosetta 2(DE3)pLysS *E. coli* strain (Millipore). The expression was induced upon consumption of the preferred glucose during culture at 17 °C for 36 h. The harvested cells were lysed by a freeze–thaw cycle in buffer-A (300 mM NaCl in 50 mM Tris-Cl, pH 7.5) containing 10 mM imidazole, 0.5 mg ml^−1^ lysozyme (Sigma) and 1 mM PMSF (Sigma). The DNaseI and MgSO_4_ were added and the solutions were transferred to Ni-Sepharose 6 Fast Flow gravity columns (GE Healthcare). The proteins were eluted with 3 ml buffer-A with 500 mM imidazole.

For HT-SELEX^17^,^39^, each E2F protein (200 ng in 3 μl each) was mixed together with DP1 protein in a 1:1 molecular ratio and mixed with DNA ligands (200–500 ng in 5 μl) containing a 6- and 3-bp barcode before and after the 40-bp randomized region in 9 μl of binding buffer (10 mM Tris-Cl, 50 mM NaCl, 1 mM MgCl_2_, 0.5 mM dithiothreitol, 0.5 mM EDTA, 4% glycerol, 5 μg ml^−1^ poly-dI-dC, pH 7.5), followed by incubation at room temperature for 20 min. Subsequently, 7.5 μl Ni-Sepharose 6 Fast Flow resin (GE Healthcare) in 142.5 μl of binding buffer was added to the protein–DNA mixture and the incubation continued for an additional 20 min. Nonspecific binding oligos were washed away with 20 volumes of binding buffer without poly-dI-dC and the complexes then suspended in 100 μl milliQ water. Finally, bound ligands were amplified by PCR (Phusion DNA polymerase) and the enriched ligands were used as input ligands for the next round of selection, which was repeated up to four times. The initial DNA library and selected ligands from each cycle were subjected to sequencing (Illumina HiSeq 2000). Position weight matrix (PWM) and adjacent dinucleotide models were generated using AUTOSEED^40^. Seeds for the PWMs shown in Fig. 3e are NTTTGGCGGGAAAN and WWWTGGCGGGAAA for E2F8 and E2F2-DP1, respectively; multinomial setting=1.

The 'riverlake' logo showing dinucleotide preferences was generated as a scalable vector graphics (svg) file using a script that draws circles for each base position in such a way that their radius is proportional to the mononucleotide frequency at that position. It then connects the circles with lines whose width is proportional to the respective dinucleotide frequency. Any observed dinucleotide frequency that is in excess of the prediction from the mononucleotide frequencies is coloured in dark blue. Any excess predicted dinucleotide frequency is indicated by yellow dotted lines. Mononucleotide frequency was calculated from the frequency of the first base of the respective dinucleotides, except for the last base, whose frequency was calculated from the frequencies of the second bases of the last dinucleotides. The E2F8 adjacent dinucleotide model is presented in Supplementary Table 2. The script is available upon request.

### ChIP-exo

LoVo (ATCC, catalogue no. CCL229TM) cells were cultured in DMEM supplemented with 10% fetal bovine serum and antibiotics. ChIP-exo was performed essentially as described in ref. 41 with modifications from Katainen *et al*.^42^ using antibodies against DP1 and E2F2, and control goat IgGs (Santa Cruz Biotechnology catalogue no.: s, sc-16286 X, sc-22821 X, sc-2028, respectively). Cells were crosslinked with 1% formaldehyde, and incubated in hypotonic buffer for 15 min, and DNA then sonicated to 200–500-bp fragments in lysis buffer (50 mM HEPES, 2 mM EDTA, pH 8.0, 150 mM NaCl, 1% Triton X-100, 0.1% sodium deoxycholate, 0.2% SDS, pH 8.0). After preclearing, lysate was subjected to immunoprecipitation overnight with the antibodies indicated (∼2.5 × 10^6^ cells in 1 ml of lysate per immunoprecipitation). The immune-complexes were precipitated using 40 μl protein G-Sepharose beads for 3 h at 4 °C, and washed successively with 0.5 ml of immunoprecipitation buffer (100 mM NaCl, 5 mM EDTA, 0.33% SDS and 1.5% Triton X-100 in 50 mM Tris-Cl, pH 8.0), 1 ml of mixed micelle buffer (150 mM NaCl, 5 mM EDTA, 5.2% sucrose, 1.0% Triton X-100 and 0.2% SDS in 20 mM Tris-Cl, pH 8.0), 1 ml of buffer 500 (250 mM NaCl, 25 mM HEPES, 0.5% Triton X-100, 0.05% sodium deoxycholate and 0.5 mM EDTA in 5 mM Tris-Cl, pH 8.0), 1 ml of lithium chloride/detergent buffer (250 mM lithium chloride, 0.5% IGEPAL CA-630, 0.5% sodium deoxycholate and 10 mM EDTA in 10 mM Tris-Cl, pH 8.0), 1 ml of TE buffer (1 mM EDTA in 10 mM Tris-Cl, pH 8.0) and 1 ml of Tris-Cl buffer (10 mM, pH 7.5, 8.0 or 9.2 according to the requirements for the enzymatic reaction steps detailed below).

Immunoprecipitates were then subjected to the following enzymatic on-bead reactions for 30 min in 60 μl reaction volume: (a) end-polishing with T4 DNA polymerase (3 U, New England BioLabs, M0203L), (b) kinase reaction with T4 polynucleotide kinase (10 U, New England BioLabs, M0201L), (c) adenine addition reaction with Klenow fragment exo-(5 U, New England BioLabs, M0212L), (d) first adaptor ligation with 1.25 μM P2 adaptors (Supplementary Table 3, rows 3 and 4; Eurofins MWG Operon) and T4 DNA ligase (500 U, New England BioLabs, M0202L), (e) fill-in reaction with phi29 DNA polymerase (10 U, New England BioLabs, M0269L), (f) lambda exonuclease reaction (10 U, New England BioLabs, M0262L) and (f) RecJf exonuclease reaction with 30 U of RecJf exonuclease (New England BioLabs, M0264L). Each reaction was set up according to the manufacturer's instructions. After each on-bead reaction, the beads were washed successively with mixed micelle buffer, buffer 500, lithium chloride/detergent buffer, TE buffer and Tris-Cl buffer. Immunoprecipitates were eluted to 400 μl of elution buffer (10 mM Tris-Cl, pH 8.0, 1 mM EDTA, pH 8.0, 400 mM NaCl, 1% SDS and 70 μg ml^−1^ RNase A), and cross-links were reversed by the addition of 20 μg of proteinase K (Thermo Fisher Scientific, EO0491) and incubation at 65 °C overnight.

Samples were then extracted with phenol:chloroform:isoamyl alcohol (25:24:1), precipitated with ethanol and processed for library preparation. Second-strand synthesis was performed using 1 μM of P2 primer (Supplementary Table 3, row 5; Eurofins MWG Operon), after which samples were denatured at 95 °C for 5 min, incubated at 58 °C for 5 min and cooled to room temperature. The primer extension reaction was performed with 10 U of phi29 polymerase, bovine serum albumin (100 μg ml^−1^) and an equimolar mixture of A, T, C and G 2'-deoxynucleoside 5′-triphosphates (dNTPs; 75 μM each) and incubation at 30 °C for 20 min. The enzyme was subsequently heat inactivated at 65 °C for 10 min. Double-stranded DNA was purified using Agencourt AMPure magnetic beads (Beckman Coulter, A63881) and eluted into 40 μl of 10 mM Tris-Cl, pH 8.0. To increase ligation efficiency, an adenine addition reaction was performed using 5 U of Klenow fragment exo- in 1 × Klenow buffer with 100 μM dATP, at 37 °C for 30 min. After DNA purification using Agencourt AMPure magnetic beads, a second adaptor ligation reaction was performed using 500 U of T4 DNA ligase, 1 × T4 DNA ligase buffer and 0.4 μM P1 adaptor (Supplementary Table 3, rows 1 and 2; Eurofins MWG Operon); samples were then incubated at 25 °C for 30 min and at 16 °C overnight. DNA was finally purified using Agencourt AMPure magnetic beads and eluted into 30 μl of 10 mM Tris-HCl, pH 8.0.

Library was PCR amplified using PCR primers with sequences provided by Illumina (PE primers; Supplementary Table 3, rows 6 and 7, Eurofins MWG Operon). PCR mix contained 2 U of Phusion High-Fidelity DNA polymerase (Thermo Fisher Scientific, F-530S), 1 × High-Fidelity Phusion polymerase buffer, 0.5 μM of each of the primers and 250 μM dNTPs in a final volume of 50 μl. PCR was carried out for 18 cycles. PCR products were size selected (200–600 bp) and gel purified using QIAquick gel purification columns (Qiagen). The purified product was sequenced at the Karolinska High-Throughput Center using the Illumina HiSeq 2000 platform according to the manufacturer's instructions. See Supplementary Table 3 for the sequences of the Illumina sequencing adaptors. Sequence reads were mapped to the hg19 human reference genome by bwa (default parameters). Peak-calling was performed using GEM^44^ with 2,700,000,000-bp genome size and default parameters. Motif discovery was performed using MEME.

### ATR-FTIR and CD experiments

FTIR spectra were recorded at 4 cm^−1^ resolution on a Vertex 70 FTIR spectrometer (Bruker Optik CpmbH, Ettingen, Germany) equipped with an HgCdTe detector. The experiments were performed with 3 μl of sample containing protein and DNA at 1:1.2 M ratio at room temperature. The absorbance spectra were recorded every 60 min for 6 h and then again after 23 h. The experiment was repeated three times. The resulting spectra were averaged and normalized to the intensity of the tyrosine band at 1,517 cm^−1^.

The CD spectra of samples containing protein and DNA at 1:1.2 M ratio were recorded on a Chirascan CD spectrometer from Applied Physics with a TC-125 temperature controller set to 20 °C. The spectral range was 178–280 nm, which required about 2 min for each scan. Each scan was performed 10 times and the average is presented.

## Additional information

**Accession codes:** The atomic coordinates and diffraction data have been deposited to Protein Data Bank with the accession code 4YO2. All sequence reads are deposited to the European Nucleotide Archive with the study accession number: PRJEB8671.

**How to cite this article:** Morgunova, E. *et al*. Structural insights into the DNA-binding specificity of E2F family transcription factors. *Nat. Commun.* 6:10050 doi: 10.1038/ncomms10050 (2015).

## Supplementary Material

Supplementary Information Supplementary Figures 1-5, Supplementary Tables 1-3 and Supplementary References

## Figures and Tables

**Table 1:** Data collection and refinement statistics.

	E2F8/P3_2_2_1_—*cr1*	E2F8/ P3_2_2_1_—*cr2*	E2F8/I23
*Data collection*			
Space group	P 3_2_ 2 _1_	P 3_2_ 2 _1_	I23
			
*Cell dimensions*			
*a*, *b*, *c* (Å)	98.83, 98.83, 121.69	99.46, 99.46, 123.38	172.82,172.82, 172.82
*α*, *β*, *γ*(°)	90, 90, 120	90, 90, 120	90, 90, 90
Resolution (Å)	14.92-3.07 (3.3-3.07)	46.13-3.76 (4.12-3.76)	54.65-3.902 (4.041-3.902)
*R*-merge	16.9 (62.4)	16.4 (74.1)	10.5 (87.7)
*I*/*σ* (I)	8.67 (1.94)	7.0 (3.17)	13.94 (1.5)
Completeness (%)	97.0 (89.2)	100 (99.7)	88.02 (74.91)
Redundancy	6.5 (6.75)	7.3 (7.3)	10.05 (3.5)
			
*Refinement*			
Resolution (Å)	14.92–3.07		
No. reflections	11,981		
*R*_work_/*R*_free_	0.27/0.29		
No. of atoms	2,130		
Protein+DNA	2130		
Average B-factor	153.70		
			
*R.m.sd.*			
Bond length (Å)	0.004		
Bond angles (°)	1.15		

Statistics for the highest-resolution shell are shown in parentheses.

Notice that only one of three data sets was used to finish structural refinement.
