# Supplementary material for: Structural insights into the DNA-binding specificity of E2F family transcription factors
Source: Nat Commun. 2015 Dec 3;6:10050. doi: 10.1038/ncomms10050 (PMC4686757; doi:10.1038/ncomms10050)
Supplement: Supplementary Information — Supplementary Figures 1-5, Supplementary Tables 1-3 and Supplementary References [file ncomms10050-s1.pdf]

Supplementary Figures

Supplementary Figure 1

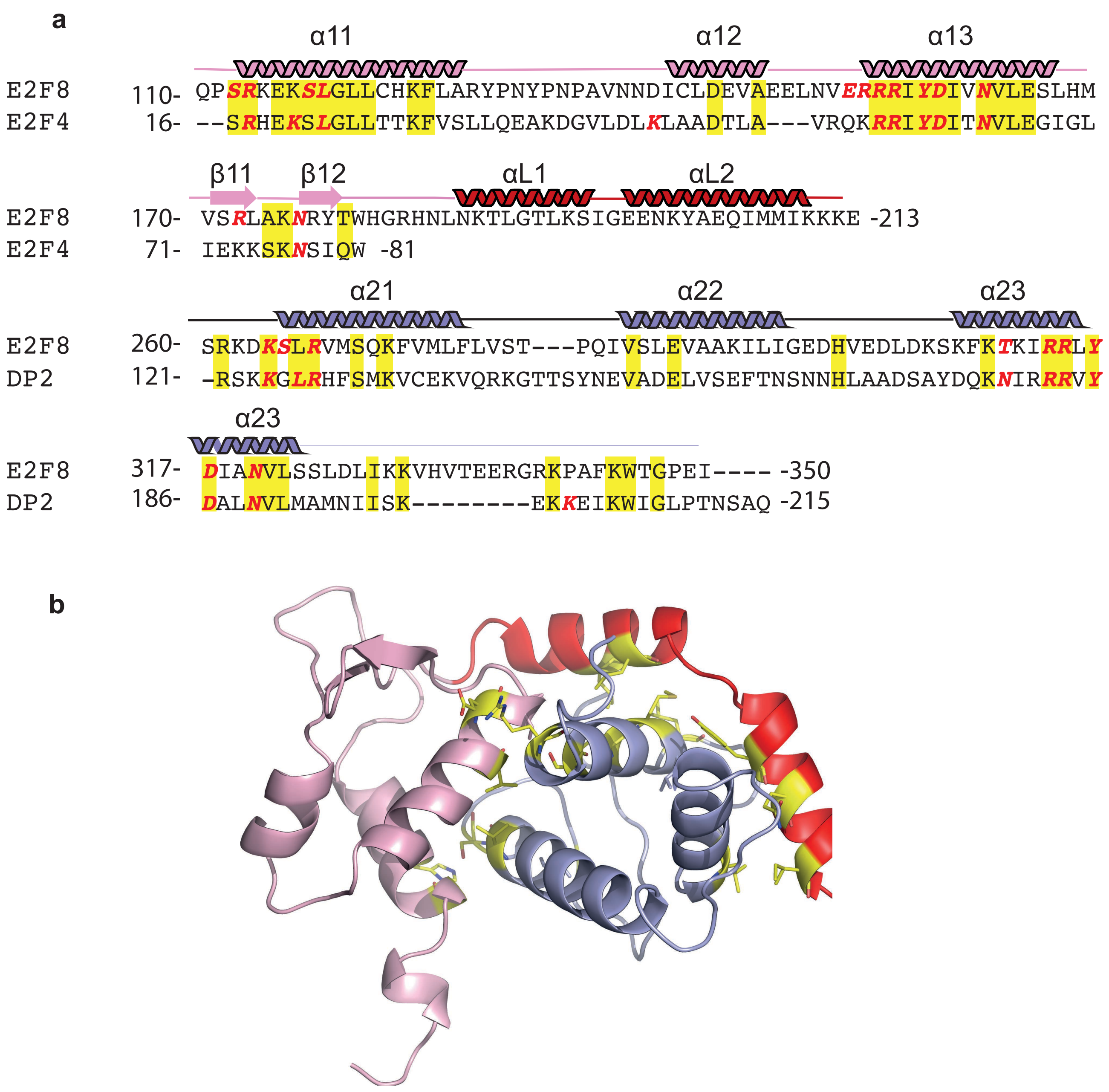

Supplementary Figure 1

E2F-DP interface in E2F8 structure. (a) Sequence alignment of the E2F8 E2F domain with the E2F4 DNA-binding domain and the E2F8 DP domain with DP2 DNA-binding domain (PDB entry 1CF7). The secondary structure found in E2F8 is presented above the sequence. The E2F8 E2F domain is in pink and the E2F8 DP domain in blue, respectively. The α-helixes of the E2F8 linking peptide are colored in red. Residues conserved throughout between E2F8 and E2F4 or between E2F8 and DP2 are highlighted in yellow; residues involved in interaction with DNA are in red bold italic. E2F8 residues are numbered according to UniProt number, E2F4/DP2 numbering is corresponding PDB entry 1CF7. (b) The representation of the E2F and DP interface: E2F domain is pink, DP domain is blue, linker is red; the residues involved in interactions are presented as ball-and-sticks and colored by atom (carbon: yellow; nitrogen: blue; oxygen: red). The residues and type of interaction are listed in Supplementary Table 1.

Supplementary Figure 2

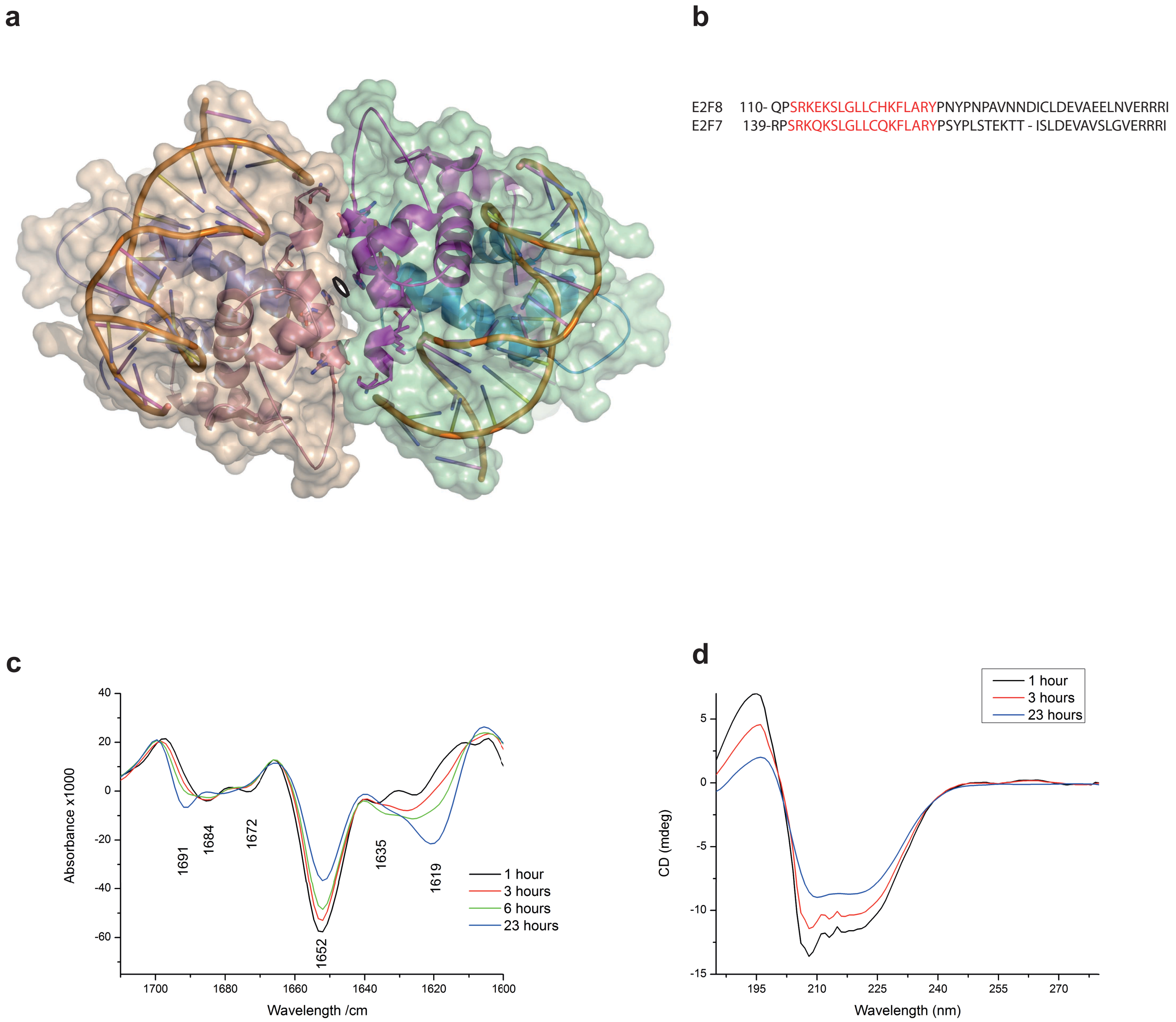

Supplementary Figure 2

(**a**) **Crystal packing contact** around the 2-fold crystallographic axis found in both, I23 and $P3_22_1$ crystal forms. Two symmetrical subunits are presented both in surface (green and gray) and cartoon, and colored separately. E2F domains are in pink, DP domains are in blue. The residues involved in contact formation are presented by sticks and colored by atoms. The two-fold axis is shown in the middle. (**b**) Sequence alignment of E2F7 and E2F8 correcponding regions involved in packing interactions.The interacting helices are colored red. (**c**) ATR-FTIR spectra of E2F8 at different time points. The position of the main band at 1652 cm^{-1} shows a small shift from typical α-helix appearance at 1650 cm^{-1}. This shift indicates the coupling of at least two parallel α-helices belonging to different molecules [1, 2, 3]. (**d**) CD spectra of E2F8: The peaks on the initial spectrum of E2F8 correspond to α-helical content of the protein. The shifts of the bands that are observed in the later spectra indicate aggregation of E2F8 [4]. ATR-FTIR and CD measurements are performed in the presence of DNA.

Supplementary Figure 3

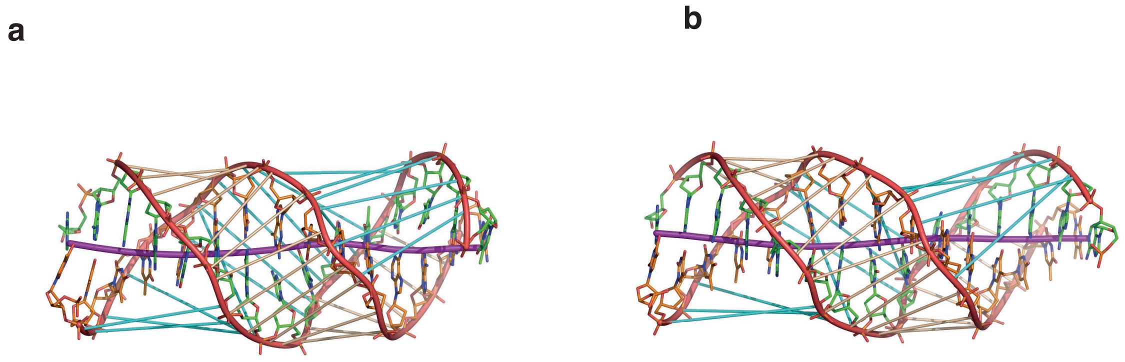

Supplementary Figure 3

DNA shape in (a) the E2F8 complex; (b) the E2F4/DP2 complex; Major and minor grooves are defined by pink and cyan vectors, respectively. The helical axes are colored in magenta.

Supplementary Figure 4

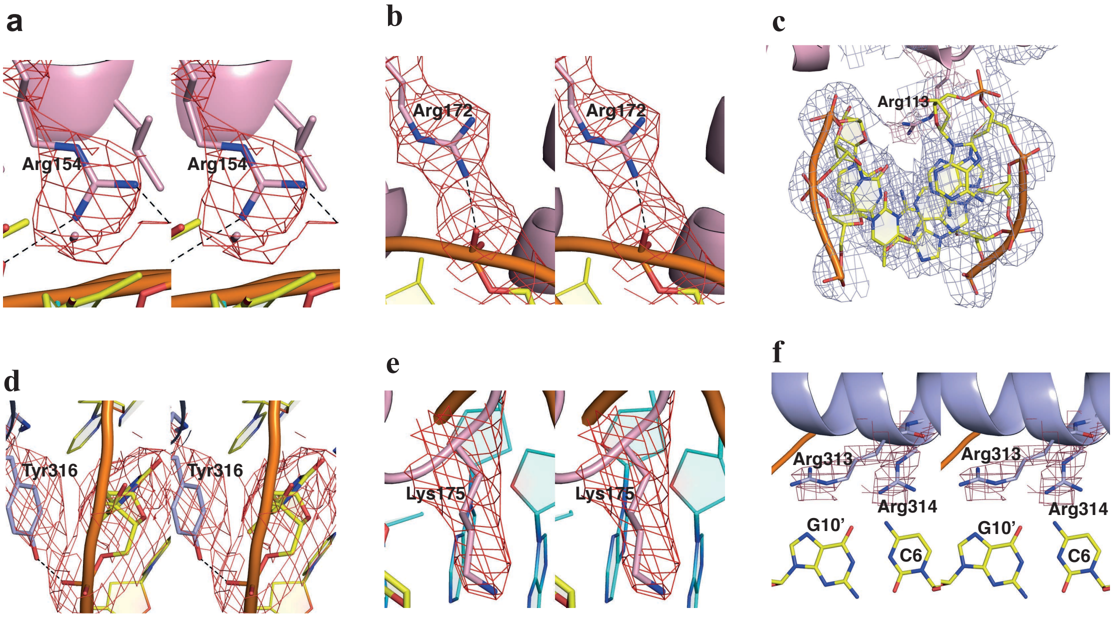

Supplementary Figure 4

Stereo images of simulating annealing omit maps 2mFo-DFc of the E2F8 structure, contoured at 1.2σ with 2.5 Å around the atoms: (**a, b**) Arg154 and Arg172 from DBD^{E2F} domain, respectively; (**c**) 2mFo-DFc map of DNA with Arg113 from N-terminus of DBD^{E2F} domain packed into the minor groove. Stereo images of simulating annealing omit maps 2mFo-DFc of: (**d**) Tyr316 from DBD^{DP} domain; (**e**) Lys175 packed in other minor groove; (**f**) Arg313 and Arg314 from binding site of DBD^{DP} domain.

Supplementary Figure 5

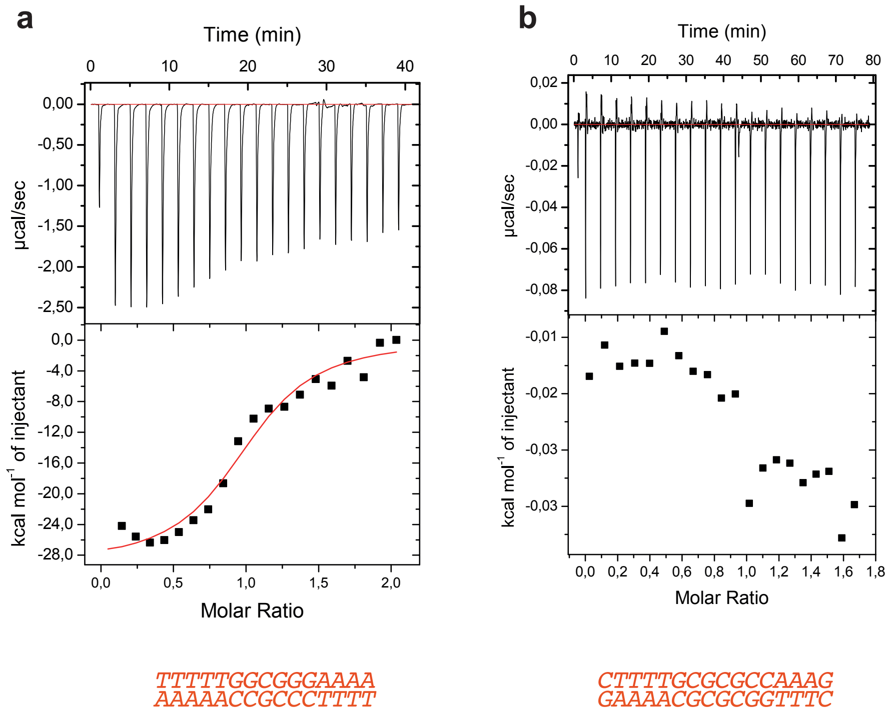

Supplementary Figure 5

Calorimetric titration profiles of E2F8 with different DNA motives. DNA sequences of binding motifs are presented under each ITC figure. The top panel of each ITC figure represents raw data; the bottom panel shows the integrated heat of the binding reaction. The red line represents the best fit to the data, according to the model that assumes a single set of identical sites. The apparent binding constant Kd is shown in the bottom panel. (a) Binding to the non-canonical non-symmetrical site enriched in HT-SELEX experiments. (b) Experiments with the canonical symmetrical site do not show any binding under the same conditions. No Kd could be determined.

Supplementary Tables.

Supplementary Table 1. Comparison of the residues involved in the DBD^{E2F}/DBD^{DP} and E2F4/DP2 contacts.

**E2F8/E2F**	**E2F8/DP**	**Type of contact**	**E2F4**	**DP2**	**Type of contact**
Gly119	Ser324	H-bond	Leu22	Val129	hydrophobic
His123	Ser324	H-bond	Val30	Met133	hydrophobic
His123	Ser324	H-bond	Asn63	Arg72	H-bond
Val163	Val321	hydrophobic	Val64	Val129	hydrophobic
Glu165	Arg267	H-bond/salt bridge	Val64	Leu71	hydrophobic
Ser166	Ser270	H-bond	Glu66	Arg72	H-bond/salt bridge
Arg172	Arg267	H-bond	Ile68	Leu130	hydrophobic
Leu190	Leu278	hydrophobic	Ile68	Leu148	hydrophobic
Leu190	Val274	hydrophobic	Leu70	Met133	hydrophobic
Leu193	Met275	hydrophobic	Lys73	Arg72	H-bond
Leu193	Val274	hydrophobic			
Ile209	Leu287	H-bond			

Supplementary Table 2. E2F8 adjacent dinucleotide model

0.319	0.406	0.204	0.030	0.010	0.233	0.050	0.109	0.424	0.551	0.718	0.488	0.299	ADM_DI	AA
0.113	0.103	0.076	0.673	0.055	0.500	0.106	0.436	0.119	0.047	0.035	0.170	0.194	ADM_DI	AC
0.173	0.146	0.247	0.286	0.927	0.100	0.794	0.236	0.305	0.173	0.184	0.153	0.205	ADM_DI	AG
0.395	0.344	0.473	0.012	0.007	0.167	0.050	0.218	0.153	0.228	0.063	0.189	0.302	ADM_DI	AT
0.262	0.302	0.155	0.030	0.002	0.080	0.000	0.100	0.016	0.910	0.346	0.305	0.214	ADM_DI	CA
0.140	0.073	0.045	0.493	0.028	0.811	0.001	0.180	0.282	0.005	0.067	0.148	0.152	ADM_DI	CC
0.344	0.288	0.366	0.472	0.967	0.064	0.998	0.560	0.647	0.056	0.222	0.312	0.400	ADM_DI	CG
0.254	0.337	0.435	0.006	0.003	0.044	0.001	0.160	0.056	0.029	0.365	0.235	0.234	ADM_DI	CT
0.225	0.286	0.117	0.027	0.001	0.004	0.151	0.000	0.002	0.896	0.469	0.428	0.184	ADM_DI	GA
0.179	0.086	0.050	0.763	0.006	0.992	0.113	0.162	0.034	0.011	0.096	0.110	0.205	ADM_DI	GC
0.235	0.227	0.119	0.202	0.993	0.002	0.594	0.836	0.942	0.041	0.144	0.114	0.289	ADM_DI	GG
0.361	0.401	0.714	0.009	0.001	0.003	0.142	0.002	0.022	0.052	0.291	0.347	0.322	ADM_DI	GT
0.195	0.201	0.106	0.027	0.040	0.270	0.084	0.015	0.159	0.781	0.514	0.274	0.201	ADM_DI	TA
0.099	0.085	0.035	0.108	0.074	0.365	0.045	0.323	0.258	0.030	0.080	0.148	0.176	ADM_DI	TC
0.189	0.108	0.160	0.855	0.856	0.216	0.742	0.446	0.357	0.066	0.159	0.204	0.242	ADM_DI	TG
0.518	0.606	0.699	0.010	0.030	0.149	0.129	0.215	0.225	0.122	0.247	0.375	0.381	ADM_DI	TT
0.314	0.249	0.305	0.160	0.028	0.002	0.004	0.001	0.001	0.003	0.775	0.677	0.446	0.244	ADM_MONO_A
0.121	0.127	0.094	0.052	0.110	0.007	0.988	0.001	0.219	0.033	0.066	0.048	0.157	0.185	ADM_MONO_C
0.231	0.213	0.176	0.231	0.850	0.990	0.003	0.996	0.776	0.938	0.060	0.170	0.159	0.258	ADM_MONO_G
0.334	0.411	0.425	0.557	0.012	0.002	0.004	0.002	0.004	0.026	0.100	0.106	0.238	0.313	ADM_MONO_T

The seed NNNNGGCGCCNNNNN was used to generate adjacent dinucleotide model (ADM)[17] for E2F8 cycle 4. The ADM is a collection of first order Markov models; conditional probabilities are indicated in the top 16 rows, and the initial probability at each position is indicated in the bottom four rows. The initial probability is given for each position to allow scoring of k-mers that are shorter than the ADM itself.

Supplementary Table 3. Sequencing adapters used for Illumina sequencing.

Adapter sequences	Direction 5' to 3'
P1 Adapter forward	ACACTCTTTCCCTACACGACGCTCTTCCGATCTNNNNT
P1 Adapter reverse	NNNNAGATCGGAAGAGCGTCGTGTAGGGAAAGAGT
P2 Adapter forward	NNNNAGATCGGAAGAGCGGTTCAGCAGG
P2 Adapter reverse	phospho-CTCGGCATTCCTGCTGAACCGCTCTTCCGATCTNNNNT
P2 primer	CTCGGCATTCCTGCTGAACC
PE forward primer	AATGATACGGCGACCACCGAGATCTACACTCTTTCCCTACACGACGCTCTTCCGATCT
PE reverse primer	CAAGCAGAAGACGGCATACGAGATCGGTCTCGGCATTCCTGCTGAACCGCTCTTCCGATCT

Supplementary References

1. Karjalainen EL, Barth A. Vibrational coupling between helices influences the amide I infrared absorption of proteins: application to bacteriorhodopsin and rhodopsin. *J Phys Chem B* **116**, 4448-4456 (2012).

2. Remorino A, Korendovych IV, Wu Y, DeGrado WF, Hochstrasser RM. Residue-specific vibrational echoes yield 3D structures of a transmembrane helix dimer. *Science* **332**, 1206-1209 (2011).

3. Ghosh D*, et al.* Structure based aggregation studies reveal the presence of helix-rich intermediate during alpha-Synuclein aggregation. *Sci Rep* **5**, 9228 (2015).

4. Micsonai A*, et al.* Accurate secondary structure prediction and fold recognition for circular dichroism spectroscopy. *Proc Natl Acad Sci U S A* **112**, E3095-3103 (2015).
